# Characterization of a Lytic Bacteriophage as an Antimicrobial Agent for Biocontrol of Shiga Toxin-Producing *Escherichia coli* O145 Strains

**DOI:** 10.3390/antibiotics8020074

**Published:** 2019-06-05

**Authors:** Yen-Te Liao, Alexandra Salvador, Leslie A. Harden, Fang Liu, Valerie M. Lavenburg, Robert W. Li, Vivian C. H. Wu

**Affiliations:** 1 Produce Safety and Microbiology Research Unit, Department of Agriculture (USDA), Agricultural Research Service (ARS), Western Regional Research Center (WRRC), Albany, CA 94710, USA; yen-te.liao@ars.usda.gov (Y.-T.L.); alexandra.salvador@ars.usda.gov (A.S.); leslie.harden@ars.usda.gov (L.A.H.); fang.liu@ars.usda.gov (F.L.); Valerie.lavenburg@ars.usda.gov (V.M.L.); 2 Animal Genomics and Improvement Laboratory, Department of Agriculture (USDA), Agricultural Research Service (ARS), Beltsville, MD 20705, USA; Robert.Li@ars.usda.gov; 3 College of Food Science and Engineering, Ocean University of China, Qingdao 266100, China

**Keywords:** STEC-specific bacteriophage, whole genome sequencing, STEC O145 strains, antimicrobial agent

## Abstract

Shiga toxin-producing *Escherichia coli* (STEC) O145 is one of the most prevalent non-O157 serogroups associated with foodborne outbreaks. Lytic phages are a potential alternative to antibiotics in combatting bacterial pathogens. In this study, we characterized a *Siphoviridae* phage lytic against STEC O145 strains as a novel antimicrobial agent. *Escherichia* phage vB_EcoS-Ro145clw (Ro145clw) was isolated and purified prior to physiological and genomic characterization. Then, in vitro antimicrobial activity against an outbreak strain, *E. coli* O145:H28, was evaluated. Ro145clw is a double-stranded DNA phage with a genome 42,031 bp in length. Of the 67 genes identified in the genome, 21 were annotated with functional proteins, none of which were *stx* genes. Ro145clw had a latent period of 21 min and a burst size of 192 phages per infected cell. The phage could sustain a wide range of pH (pH 3 to pH 10) and temperatures (−80 °C to −73 °C). Ro145clw was able to reduce *E. coli* O145:H28 in lysogeny broth by approximately 5 log at 37 °C in four hours. These findings indicate that the Ro145clw phage is a promising antimicrobial agent that can be used to control *E. coli* O145 in adverse pH and temperature conditions.

## 1. Introduction

Shiga toxin-producing *Escherichia coli* (STEC) is a notorious foodborne pathogen that can cause severe illness, such as hemolytic uremic syndrome (HUS), which has a high mortality rate among young children and the elderly [1]. The first known STEC outbreak occurred in 1982 and was associated with the consumption of undercooked hamburger patties contaminated with *E. coli* O157:H7 strains [2]. Since then, the number of STEC-related infections, including the serogroups of O157 and non-O157 with O26, O45, O103, O111, O121, and O145 in particular, has increased every year, with an estimated 176,000 cases, 2400 hospitalizations, and 20 deaths annually in the U.S. [3]. In recent years, foodborne illnesses associated with consuming contaminated produce have increased [4]. STEC is one of the most frequently-occurring bacterial pathogens, responsible for about 18% of produce-associated outbreaks in the United States [5]. Next to STEC O157, STEC O145 is the most widespread pathogen among the top six non-O157 STEC serogroups associated with human infections in the U.S. [6,7]. In 2010, STEC-O145-contaminated romaine lettuce led to a serious foodborne outbreak in multiple states [8]. Infections caused by STEC O145 strains have been reported around the world [9,10]. 

To trace the contamination source, Carter et al. compared various STEC O145 strains isolated from different environmental sources that had previously been implicated in produce-associated outbreaks including animal feces (cattle and pigs), sediment, surface water around produce-growing regions, and other environmental sources [11]. The authors found that although these strains belonged to the same serogroup (O145), they had significant phenotypic variation that could be associated with natural selection as a result of exposure to different environmental stresses. The changing phenotypic characteristics of these pathogens and/or the development of antimicrobial resistance could challenge existing antimicrobial interventions. A previous study revealed that several weak acids that were effective against *E. coli* O157:H7 were insufficient to control non-O157 STEC strains [12]. Therefore, alternative approaches are needed to prevent the spread of these pathogens.

Bacteriophages (or phages) are some of the most highly diverse and abundant entities in the biosphere, being approximately 10 times more prevalent than bacteria [13]. Phages may have different associations with their bacterial hosts primarily due to two different infection cycles: lytic and lysogenic [14]. Due to concerns surrounding antibiotic resistance as well as advantages of host specificity of lytic phages against the bacterial hosts, interest in isolating and characterizing various lytic phages is growing to use phages as an alternative to antibiotics in controlling bacterial pathogens [15]. Another study evaluated the effectiveness of seven phages isolated from different sources on the reduction of *E. coli* O157:H7 in a post-harvest setting [16]. The authors found that the most promising phage, with strong lytic effects, was isolated from municipal wastewater; it resulted in approximately 2.5 and 3.5 log colony forming unit (CFU)/g reduction in *E. coli* O157:H7 on cut green pepper and spinach leaves after phage treatment, respectively. Amarillas et al. isolated and characterized a phage from horse feces that was capable of infecting multidrug-resistant *E. coli* O157:H7 strains and some *Salmonella* strains [17]. Another study isolated two T5-like phages from food samples; these phages were able to infect different species of pathogens including STEC O152 and O103, *Shigella sonnei*, *Salmonella*, multi-drug resistant *E. coli*, and generic *E. coli* strains [18]. Tolen et al. evaluated the effectiveness of current available prototype bacteriophage intervention on the reduction of STEC O157 and non-O157 strains inoculated on cattle hide [19]. However, none of these commercial phage products targets STEC O145 strains.

The studies discussed above primarily focused on the antimicrobial activities of phages targeting either *E. coli* O157:H7 or other antibiotic-resistant *E. coli* strains; similar information regarding phages that are lytic against non-O157 STEC is relatively scarce. Therefore, the objective of this study was to characterize a bacteriophage isolated from non-fecal compost and to examine its potential as a novel biocontrol agent for STEC O145 strains.

## 2. Results 

### 2.1. Genomic Analyses of Ro145clw

In this study, *Escherichia* phage vB_EcoS-Ro145clw (also known as Ro145clw) was isolated from non-fecal compost samples using *E. coli* O145 (RM10808) as the primary host strain. After purification, the extracted phage DNA was sequenced; the assembled phage genome had 4350× coverage. Phage Ro145clw has a 42,031 bp double-stranded DNA and an average G + C content of 50.6%. The BLASTn results showed that both *Escherichia* phage K1G (GenBank accession #GU196277) and *Escherichia* phage P AB-2017 (GenBank accession #KY295898), belonging to the family *Siphoviridae,* had the highest nucleotide similarity to phage Ro145clw. Further JSpeciesWS analysis revealed that Ro145clw shared an 84.05% and 83.58% average nucleotide identity based on BLAST (ANIb) with K1G and P AB-2017 phages, respectively. These results indicated that the Ro145clw genome belongs to the *Siphoviridae* family. 

Genome annotation predicted 67 putative open reading frames (ORFs), of which 21 encoded functional proteins that were associated with phage DNA replication, packaging, structural proteins, and host cell lysis (Supplementary, Table S1). The six annotated ORFs in the Ro145clw genome associated with DNA replication included putative thermostable DNA polymerase I, putative helicase, transcriptional repressor DicA, putative helicase-primase, putative PD-(D/E)XK nuclease superfamily protein, and putative calcineurin-like phosphoesterase superfamily domain protein. At least nine predicted ORFs in Ro145clw were annotated as phage structural proteins in Ro145clw, including tail protein, tail assembly chaperone protein, tail fiber, head protein, major capsid protein, decoration protein, and tape measure protein. Three consecutive ORFs in Ro145clw (ORF_51, ORF_52, and ORF_53) coded for putative holin-like class II, holin-like class I, and endolysin, respectively, were associated with forming a holin-dependent host cell lysis system [20]. Both ORF_51 and ORF_52 shared 92% and 95% average nucleotide identity, respectively, with their counterparts in phage G AB-2017, whereas ORF_53 shared 86% average nucleotide identity to the gene encoding lysozyme in *Escherichia* phage P AB-2017 [21]. Two spanin proteins encoded by ORF_66 and ORF_67 were located downstream of the cell lysis system in the Ro145clw genome and were associated with the final step of cell lysis by breaking the structure of the outer membrane of the host cell to release the phage progenies [22]. No virulence genes (such as *stx*, antibiotic-resistance genes, or tRNAs) were found in Ro145clw. PhageTerm analysis predicted that phage Ro145clw had a headful DNA packaging mechanism with a preferred packaging (*pac*) site [23].

**Figure 1:**
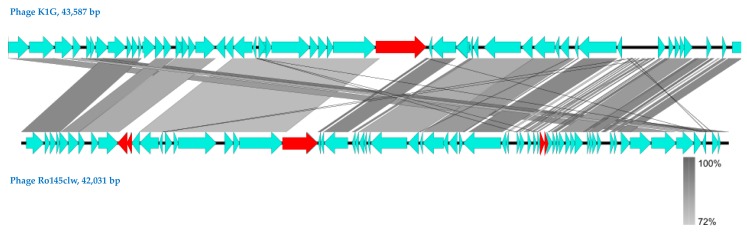
Genome comparison of Ro145clw and its reference phage K1G, using BLASTn and visualization with EasyFig. Genome maps of phages K1G and Ro145clw are presented as turquoise blue arrows, which indicate the order of annotated open reading frames (ORFs) from left to right along the phage genomes. Regions of sequence similarity are connected by a gray-scale shaded area, and the unshared ORFs are highlighted in red. Capital letters indicate the ORFs associated with (A) putative tail assembly chaperone, (B) tape measure, (C) putative structural, (D) tail, and (E) major capsid protein observed on the SDS-PAGE gel (refer to Figure 6).

**Figure 2:**
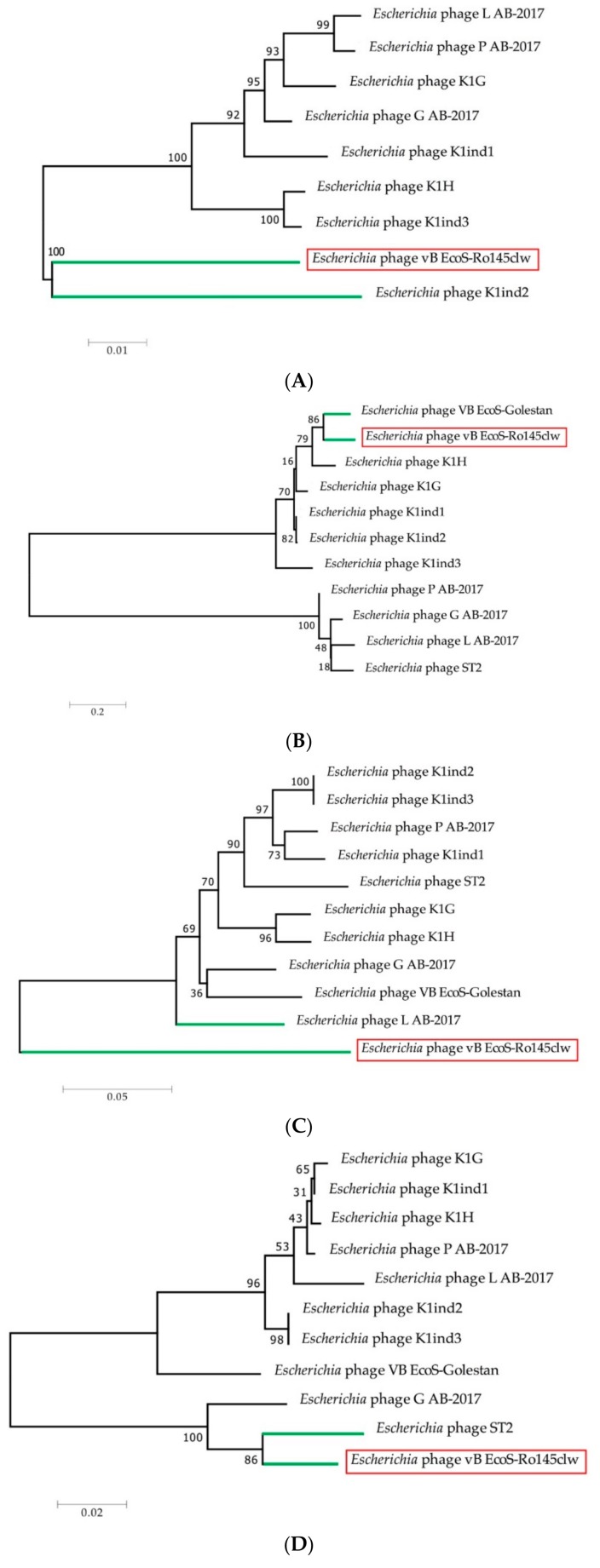

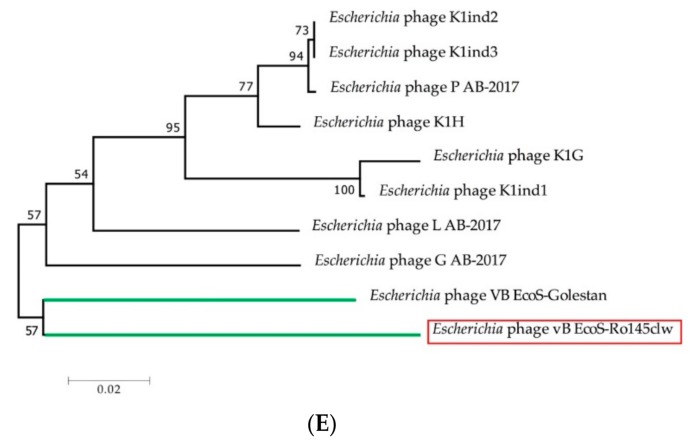
Neighbor-joining phylogenetic tree of phage vB-EcoS-Ro145clw (highlighted with a red box) and the closely-related K1glikevirus reference genomes based on the Clustal Omega alignment of the sequences of (**A**) terminase, (**B**) tail protein, (**C**) tape measure, (**D**) holin-like class I, and (**E**) endolysin. Numbers next to the branches are bootstrap values (500 replicates). The scale represents the homology percentage. Green lines are used to indicate the closest evolutionary relationship between the reference phages and phage vB-EcoS-Ro145clw.

Comparative analysis showed that phages Ro145clw and K1G shared similar ORF content (Figure 1); however, one ORF-encoding tailspike protein that was present in K1G was absent in Ro145clw. One putative tail fiber protein encoded by ORF_21 and four hypothetical proteins encoded by ORF_10, ORF_11, ORF_45, and ORF_46 in the Ro145clw genome were absent in the K1G phage genome (Figure 1). Phylogenetic analysis of terminase showed that Ro145clw was closely related to the phage K1ind2 (Figure 2A), indicating a similar headful packaging strategy. The analysis of both genes encoding tail and endolysin indicated that Ro145clw had a close evolutionary relationship with phage VB EcoS-Golestan (Figure 2B,E). The genes encoding tape measure protein and holin-like class I in Ro145clw showed maximum similarity with the counterparts in phage L AB-2017 (Figure 2C) and phage ST2 (Figure 2D), respectively.

### 2.2. Morphology and Host Range of Ro145clw

**Figure 3:**
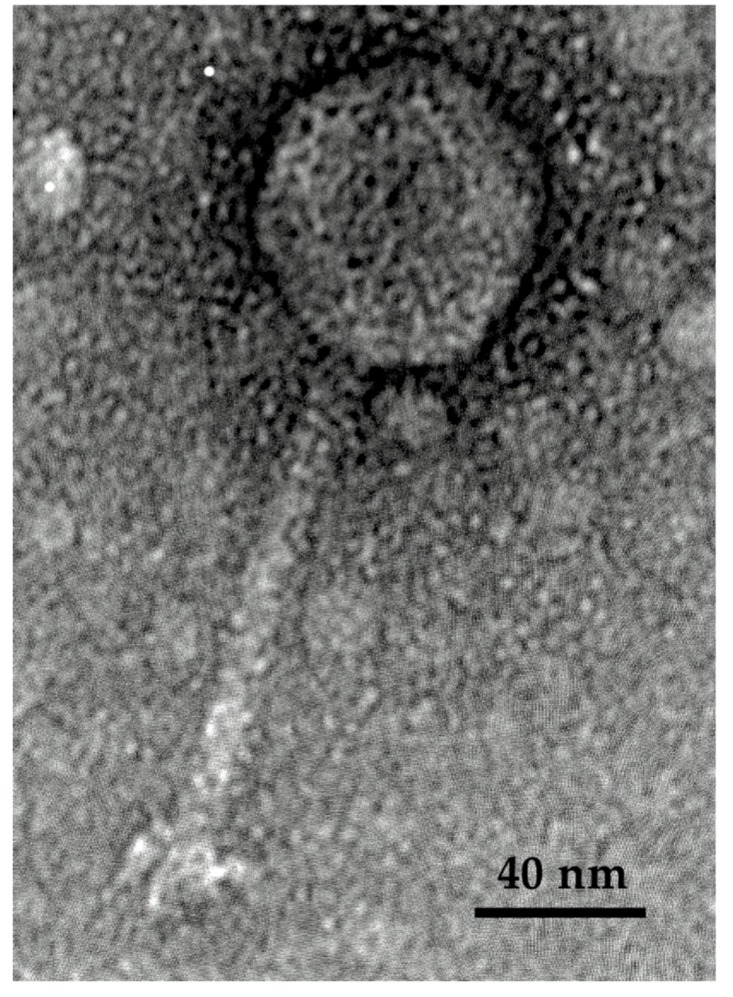
Transmission electron microscopy image of phage Ro145clw with a long and non-contractile tail, showing *Siphoviridae* morphology.

Phage Ro145clw displayed a morphology containing an icosahedral head approximately 58–62 nm in diameter and a long non-contractile tail 122.7 ± 2.5 nm in length, which is typical of phages belonging to the *Siphoviridae* family (Figure 3). Ro145clw contained a base plate structure that resembled a rosette with three to four leaves, with an estimated diameter of 27.3 ± 2.5 nm.

The results of the spot test assay indicated that phage Ro145clw is able to produce a lysis zone on the selected STEC O145 strains, with complete lysis against all the environmental STEC O145 strains, but incomplete lysis against the outbreak strains including RM13514, RM13516, RM12581, and RM12761 (Table 1). Efficiency of plating (EOP) was used to determine the productive infection of the test strains in comparison with the primary strain used for phage isolation. The results showed that the *E. coli* O145:NM strain had a high phage-producing efficiency similar to that of the primary host strain, and four *E. coli* O145 strains (RM8732, RM11691, RM9872, and RM12367) had high phage-producing efficiency (EOP > 0.5) after phage Ro145clw infection (Table 1). One *E. coli* O145:H-strain had medium phage-producing efficiency, and four *E. coli* O145:H28 strains resulted in inefficient phage production (EOP < 0.001).

### 2.3. One-Step Growth Curve

**Figure 4:**
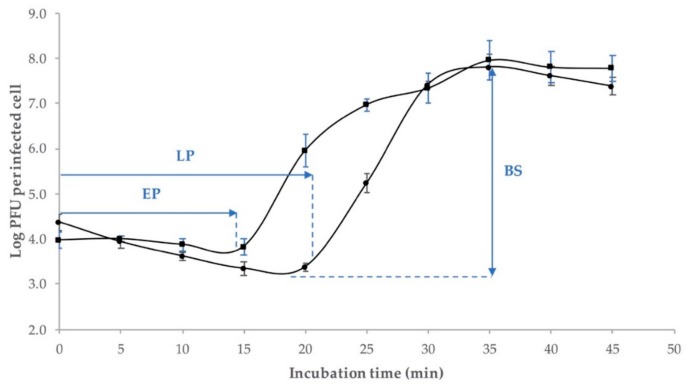
One-step growth curve of the phage Ro145clw using *E. coli* O145 strain (RM10808). The growth parameters of the phage indicate an eclipse period (EP) of 14 min, a latent period (LP) of 21 min, and an average burst size (BS) of 192 phages per infected cell. Closed circles indicate non-chloroform-treated samples; closed squares indicate chloroform-treated samples. The error bars present the standard error of the mean for each time point of the one-step growth curve.

The growth factors of phage Ro145clw, including the eclipse period, latent period, and burst size, were evaluated. The results demonstrated that Ro145clw phage had an approximately 14-min-long eclipse period and a 21-min-long latent period (Figure 4). An average burst size of 192 phages per infected cell was observed at approximately 35 min of incubation at 37 °C (Figure 4).

### 2.4. Phage pH and Temperature Stability

**Figure 5:**
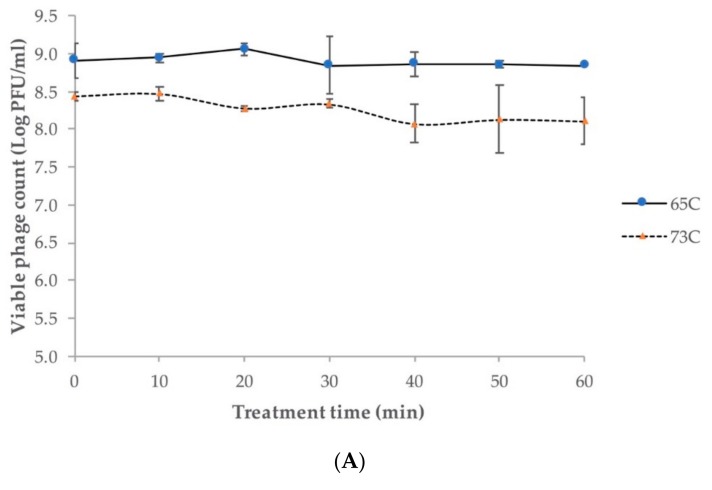

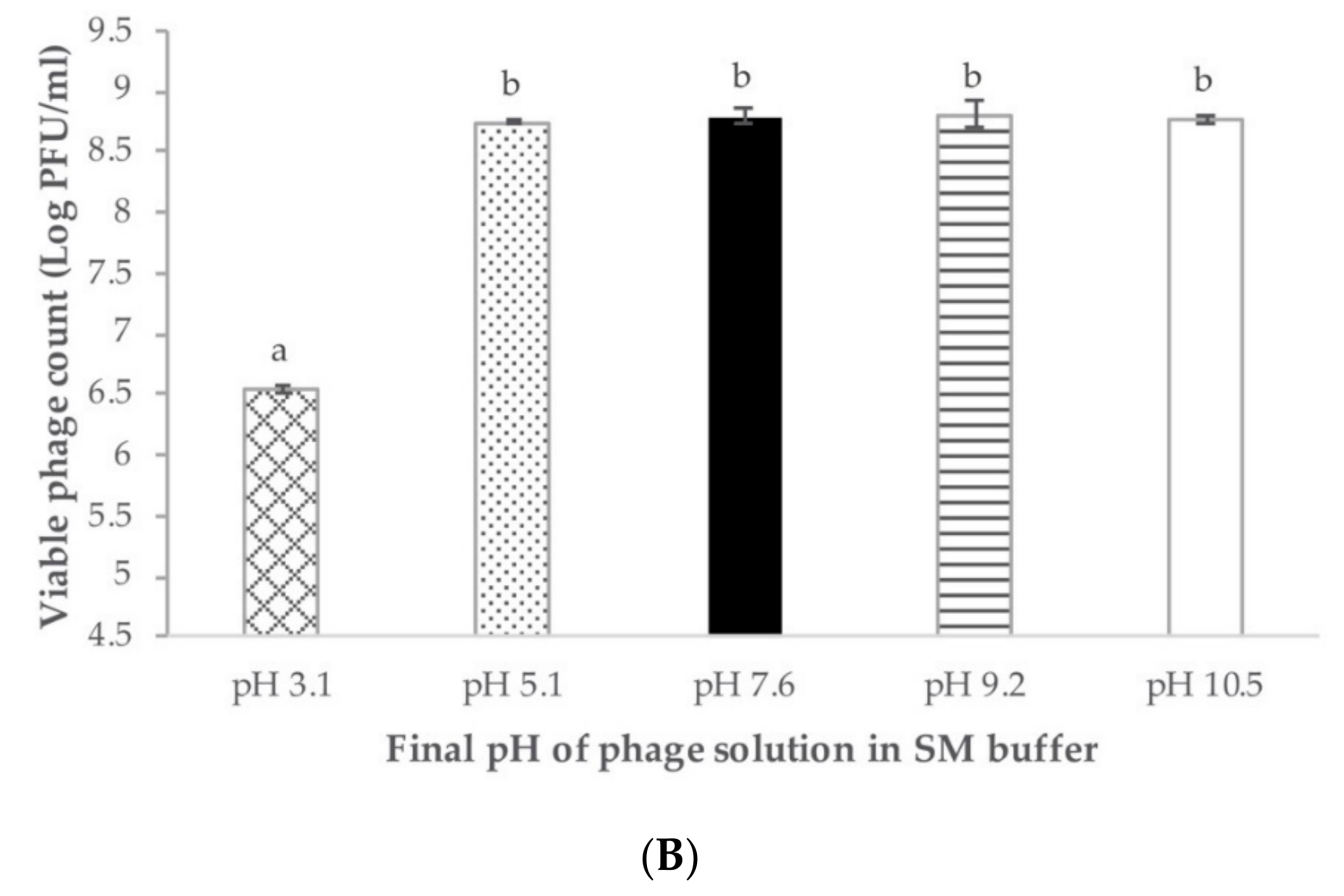
Stability of phage Ro145clw at (**A**) different temperatures (65 °C and 73 °C) for one hour and (**B**) various final pH (pH 3.1, pH 5.1, pH 7.6, pH 9.2, and pH 10.5) for 24 h. No statistical differences were observed between each time point of the thermal stability (*p* > 0.05). For the pH stability test, means of phage titers from different pH treatments that lack common letters (a and b) differ (*p* < 0.05). SM buffers with the initial pH of 2.2, 4.5, 7.5, 10, and 12 were used for the pH test. The error bars show the standard error of the mean (SEM).

Regardless of the different initial phage concentrations for treatment, Ro145clw was stable at 65 °C and 73 °C during the one-hour investigation, with only 0.1 and 0.3 log plaque-forming unit (PFU)/mL reductions in phage titers, respectively (Figure 5A). The phage stock with an initial concentration of 1 × 10^10^ PFU/mL in 25% glycerol remained at a similar titer when stored at –80 °C for five months (Supplementary, Table S2). Regarding pH stability, the results showed that phage Ro145clw maintained similar titers (*p* > 0.05) in a range of final pH from 3.1 to 10.5 after incubation at 37 °C for 24 h (Figure 5B). However, the phage titer was significantly reduced at pH 3.1 by 2.2 log PFU/mL compared to other pH treatments (*p* < 0.05). The results indicated that phage Ro145clw was able to be sustained in a wide pH range.

### 2.5. Analysis of Phage Structural Proteins

**Figure 6:**
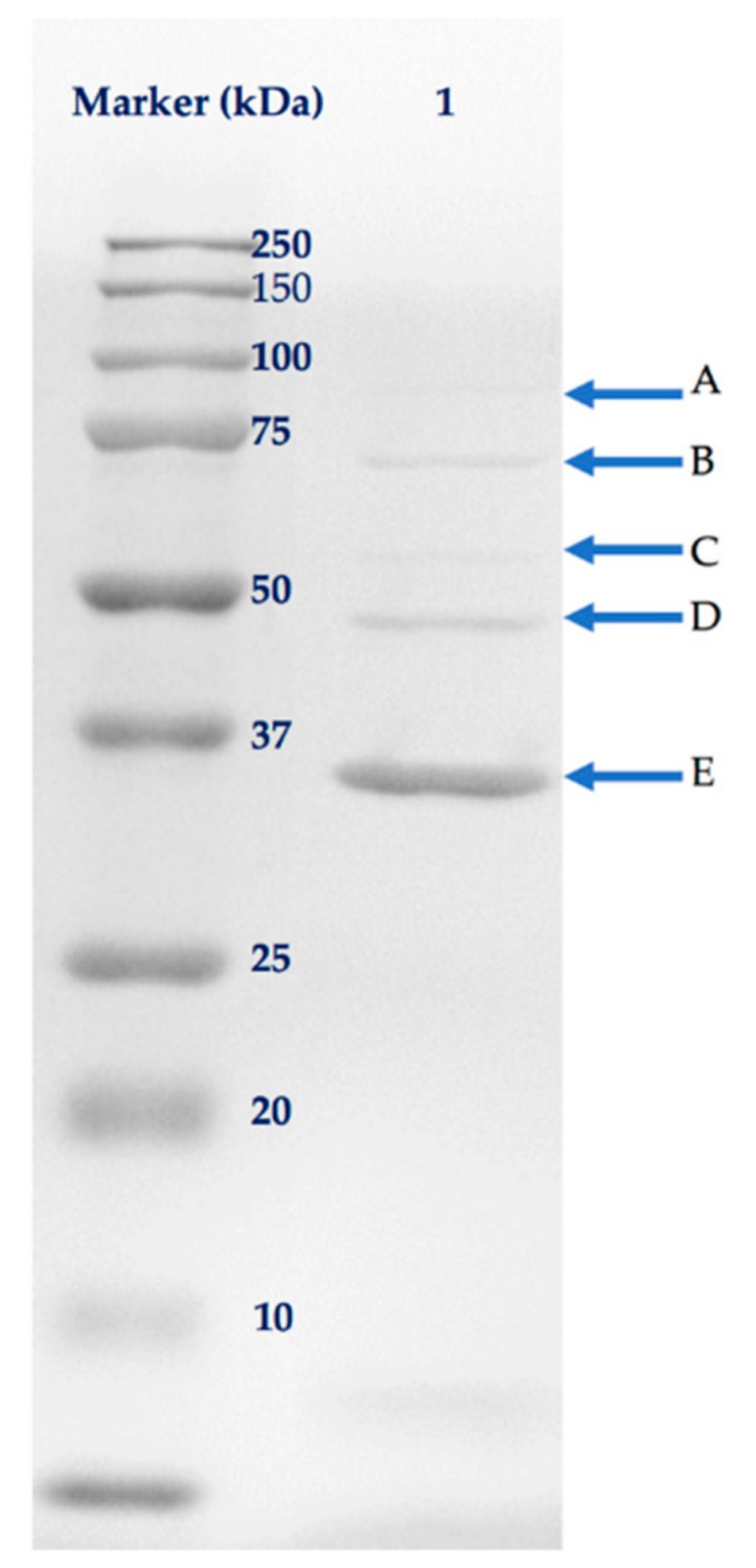
The structural proteins of phage Ro145clw (lane 1) on a 12% SDS-PAGE gel, visualized by Coomassie brilliant blue R-250. A = Putative tail assembly chaperone; B = Tape measure protein; C = Putative structural protein; D = Tail protein; E = Major capsid protein.

The separation of phage proteins by sodium dodecyl sulfate-polyacrylamide gel (SDS-PAGE) revealed five bands related to phage structural proteins, with molecular weights ranging from approximately 37 to 100 kDa (Figure 6). The identified structural proteins included putative tail assembly chaperone, tape measure protein, phage structural protein, tail protein, and major capsid protein, with coverage of amino acid sequences ranging from 11 to 62% by mass spectrometry (Table 2). Five out of eight structural proteins predicted from the genomic data were identified by mass spectrometry. None of the proteins associated with DNA replication or host lysis were detected in the SDS-PAGE gel. 

### 2.6. Antimicrobial Activity against E. coli O145:H28 in Lysogeny Broth (LB)

The effect of the multiplicity of infection (MOI) of phage Ro145clw on the growth of the *E. coli* O145:H28 strain was monitored in 96-well plates using a spectrophotometer prior to the in vitro bacterial reduction study in LB. Regardless of MOI, the bacteria of all treatment groups started to grow in a similar pattern to the control group before four hours of incubation at 37 °C (Supplementary, Figure S1). However, in contrast to the control, bacterial growth was suppressed (not a prompt decrease) by the treatment of phage Ro145clw; regardless of the MOIs, this was first observed at approximately five hours’ incubation, and continued with minimum growth throughout the experiment period (Supplementary, Figure S1). Due to similar bacterial suppression between the treatments of MOI 10 and MOI 100, a MOI of 100 was selected for the in vitro antimicrobial study in LB.

**Figure 7:**
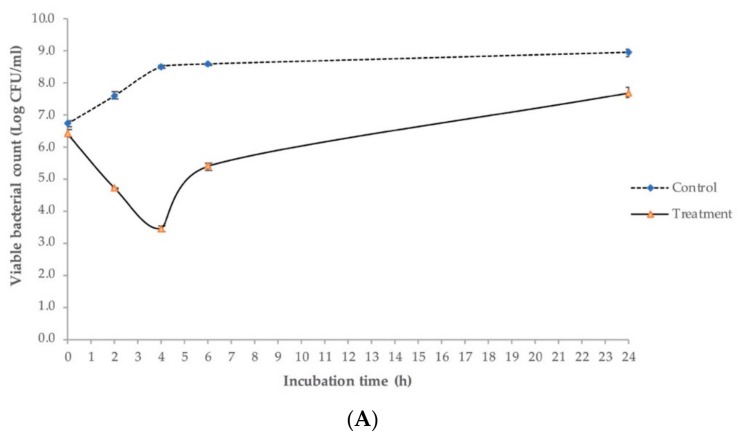

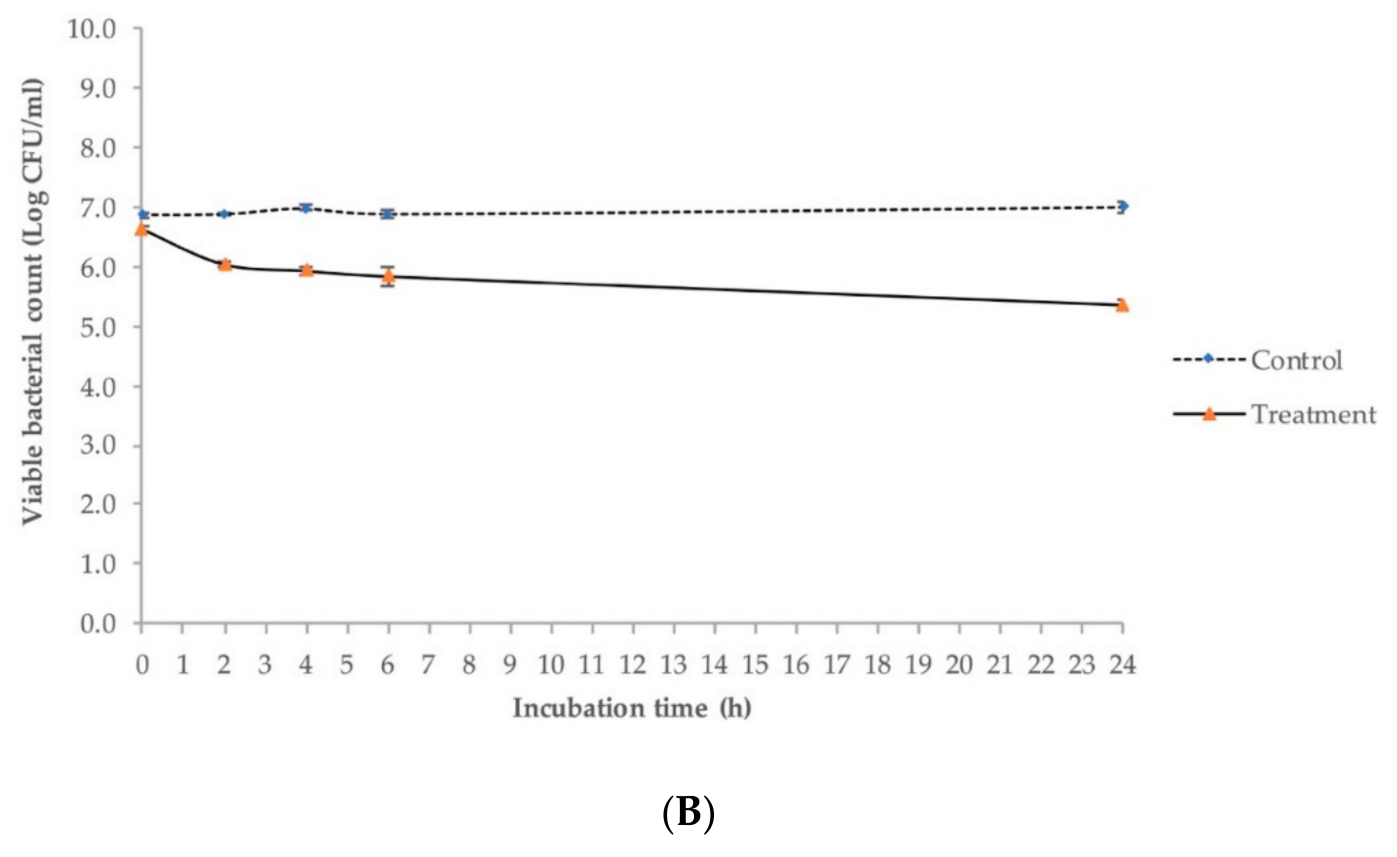
Antimicrobial effects of phage Ro145clw (multiplicity of infection (MOI) of 100) on *E*. *coli* O145:H28 (RM13514) in LB at (**A**) 37 °C and (**B**) 8 °C for 24 h. The control group contained bacterial culture without phages (dashed line) and the treatment group contained bacterial culture treated with phage Ro145clw (solid line). The error bars present the SEM for each time point of the treatment.

The in vitro antimicrobial effects of Ro145clw against the *E. coli* O145:H28 strain at different temperatures are illustrated in Figure 7. At 37 °C, the culture of *E. coli* O145:H28 without phage treatment (control) increased 0.8 log after two hours of inoculation and reached 9 log CFU/mL after 24 h of incubation. In the treatment group using phage Ro145clw, *E. coli* O145:H28 levels showed significant decreases of 2.87, 5.07, and 3.20 log (*p* < 0.05) in comparison to the control at two, four, and six hours, respectively (Figure 7A). Although the phage-treated culture commenced growing after 4 h of incubation, viable *E. coli* O145:H28 cells were still reduced by 1.25 log (*p* < 0.05) less than the control group after 24 h (Figure 7A). The treated *E. coli* O145:H28 was reduced by 3.51 and 1.05 log after six hours and 24 h of incubation at 25 °C, respectively (Supplementary, Table S3). At 8 °C, the phage treatment resulted in reductions in *E. coli* O145:H28 by 0.82, 1.15, 1.17, and 1.74 log compared to the control group at two, four, six, and 24 h, respectively (Figure 7B). The phage was able to reduce the *E. coli* O145:NM strain with high EOP by approximately 2.7 log more than the reduction of the low EOP strain, *E. coli* O145:H28, after six hours incubation at 25 °C (Supplementary, Table S3). Additionally, the bacteriophage-insensitive mutant (BIM) frequency was 8.70 ± 1.22 × 10^−2^ for *E. coli* O145:H28 strain and 3.52 ± 2.27 × 10^−5^ for *E. coli* O145: NM strain. As expected, phage Ro145clw was more common against environmental STEC O145 strain than the outbreak strain. 

## 3. Discussion

Increasing numbers of foodborne outbreaks have been associated with food contaminated by non-O157 STEC. Although phages have been studied for their potential to control bacterial pathogens, the number of commercially-available phages and published studies on phages that are lytic against non-O157 STEC are relatively few in comparison with those on STEC O157. In the current study, a new phage, vB_EcoS-Ro145clw (or Ro145clw), which produces lytic activity against STEC O145 strains, was isolated from a non-fecal environment, unlike the majority of STEC-specific phages that have been isolated from animal-associated environments such as feedlots or fecal samples [24,25]. Though the prevalence of STEC-specific phages is highly related to the presence of their STEC hosts, which are commonly found in ruminant feces, our previous study demonstrated that various STEC-specific phages could also be isolated from produce-growing areas in Salinas Valley, CA, U.S. with STEC O145-specific phages being the most prevalent [26]. 

For biocontrol applications to be developed, knowledge of the genome sequence of lytic phages is critical for ensuring that no lysogenic factors, virulence-related genes, or antibiotic-resistance genes are encoded [27]. The phage Ro145clw contains a 42,031-bp double-stranded DNA genome with an average G + C content of 50.6%, but no tRNA. There are no unwanted genes such as lysogenic genes, *stx*, *eae*, or antibiotic resistance genes present in the Ro145clw genome, which makes the phage suitable for biocontrol uses. Comparative analysis showed that the phage Ro145clw is closely related to a group of phages belonging to the genus *K1gvirus*, with 84.05% BLAST-based ANI over 64% of the aligned K1G phage sequences. No *K1gvirus* phages encode tRNA, and they all have average genome sizes ranging from 42 to 46 kb, with G + C contents over 50%. This evidence suggests that phage Ro145clw might belong to the genus *K1gviru*, according to the Bacterial and Archaeal Viruses Subcommittee description [28]. Phylogenetic analysis indicates that different core genes in the Ro145clw genome have a close evolutionary relationship with different *K1gviru* phages, most likely due to horizontal genetic transfer during the evolutionary process [29,30]. 

Transmission electron microscopy (TEM) showed that the phage Ro145clw morphology belongs to the family *Siphoviridae*; this result is consistent with the taxonomic classification based on the BLASTn search of the genome. The phage contains a unique rosette-like base plate structure, which is not commonly seen in siphophages, but has been previously described in Rtp [31] and KLPN1 [32] phages. However, the actual function of the structure has not been well elaborated. The host range of Ro145clw is narrow and STEC-O145-specific. The results of this study indicate that environmental STEC O145 strains are more susceptible to phage Ro145clw infection than outbreak strains. The phage has a latent period of 21 min and a burst size of 192 phages per infected cell, which is considered to be a large burst size in comparison with other coliphages such as phiC119 (210 phages per infected cell) and vB_EcoS-B2 (224 phages per infected cell) that are lytic against different *E. coli* strains [17,33]. A previous study demonstrated that a phage with a large burst size is favorable as a biocontrol agent because the phage population could substantially multiply within a short period of time to control the target bacterial cells [34]. A complete cycle of Ro145clw infection against the STEC O145 strain used for isolation took approximately 45 min, which was approximately 20 min and 30 min shorter than those of phage phiC119 [17] and phage PE37 [35], respectively, against *E. coli* O157:H7 strains. 

These findings suggest that phage Ro145clw has a relatively short infection cycle against its host strain. Wang et al. isolated a phage, phage AYO145A, that was lytic against STEC O145 strains from bovine feces in Canada [24]. The phage belongs to the family *Myoviridae* and has a narrow host range. Although whole genome sequencing was used to unveil the genomic characters of AYO145A [36], information regarding further physiological characterization, such as the one-step growth curve and the antimicrobial activity of the phage, is lacking. Another study evaluated a number of phages that were lytic against different serogroups of non-O157 STEC that had been isolated from cattle feces in the United States [25]. Their results showed that four myophages that were lytic against STEC O145 had different burst sizes, ranging from 23 to 195 phages per infected cell, and were also able to infect STEC O111 strains. However, a genomic characterization of these phages was not conducted and thus the presence of unwanted genes such as *stx* could not be determined. To the best of our knowledge, our current study is the first to provide both physiological and genomic information regarding a phage specific to STEC O145 strains.

Phage stability is critical and closely associated with the effectiveness of controlling pathogens during application. Previous studies indicate that each phage responds differently to exposure to external stress [25,37]. The results of this study indicate that phage Ro145clw is resistant to a pH change from 5.1 to 10.5, but a reduction of approximately 2.2 log was observed at pH 3.1. Ro145clw is able to withstand pasteurization temperatures (63 and 73 °C), and the thermal stability feature of the phage is likely to be associated with the gene encoding tape measure protein [38]. Son et al. found that a STEC O157-specific phage (isolated from bovine intestine) was susceptible to pH changes, with 1 log PFU/mL reductions at either pH 6 or 8 [35]. Litt et al. found that most phages were able to survive in a pH range from 5 to 9, but dropped off significantly when the pH went higher or lower than this range [25]. The findings of this study indicate the potential utility of phage Ro145clw in a wider range of conditions.

The genomic and biological characterization of phage Ro145clw indicates that the phage has a biocontrol potential for STEC O145 strains. The antimicrobial activities of the phage against an outbreak strain (*E. coli* O145:H28) were evaluated in LB in this study. The results showed that the strain was reduced by 5.07 log after 4 h of incubation at 37 °C and commenced growing for the remaining incubation time. Though the difference was not significant, phage Ro145clw was more active in reducing *E. coli* O145:H28 at 25 °C than at 37 °C during the first quarter of incubation period. However, at 8 °C, the antimicrobial activity of phage Ro145clw decreased. Son et al. reported a similar trend, showing that the phage reduced higher levels of *E. coli* O157:H7 at 25 °C than at 8 °C in LB [35]. The authors also found that phage-treated *E. coli* O157:H7 culture started to grow after 6 h of incubation at room temperature. This trend has been observed in other studies, as the time required for the treated bacterial culture to grow varies from strain to strain [39,40]. However, the mechanisms associated with the phage-treated bacteria’s regrowth require further investigation. The antimicrobial activities of phage Ro145clw resulted in a greater reduction in environmental STEC O145 strains than the outbreak STEC O145 strain (RM13514) at room temperature (25 °C). These findings suggest that phage Ro145clw is a suitable biocontrol agent for STEC O145 in pre-harvest environments, such as those present in produce farms. 

## 4. Materials and Methods

### 4.1. Bacterial Strains Preparation

Three non-pathogenic *E. coli* strains and 14 STEC strains were selected as host strains for the isolation of STEC-specific phages in this study. The *E. coli* strains included ATCC13706, ATCC43888, and DH5α, and the STEC strains included two strains per serogroup of O26, O45, O103, O111, O121, O145, and O157 STEC. These were obtained from the culture collection of the Produce Safety and Microbiology (PSM) Research Unit at the US Department of Agriculture (USDA), Agricultural Research Service (ARS), Western Regional Research Center, Albany, CA, USA. Seven additional STEC O145 strains, including four outbreak strains and five *Salmonella* strains (also obtained from the PSM culture collection), were used to test the antimicrobial activity of the phage (Supplementary, Table S4). All STEC strains were previously isolated from different environmental samples, such as water and animal feces, and were further confirmed with the presence of *eae* and *stx* genes. Fresh cultures of the strains were prepared by inoculating a sterile 5 mL tryptic soy broth (TSB; Difco, Becton Dickinson, Sparks, MD USA) with a loopful of the individual strain, and were incubated overnight at 37 °C prior to use. 

### 4.2. Bacteriophage Isolation

Non-fecal compost samples, derived from composted food scraps and yard trimmings, were collected from a composting operation. For phage isolation, the samples were enriched with a 14-strain STEC cocktail and a 3-strain non-pathogenic *E. coli* cocktail in TSB, supplemented with 10% calcium chloride solution at 37 °C overnight. After centrifugation at 8000× *g*, the supernatant was obtained and used to confirm the presence of STEC-specific phages using a spot test assay against each individual strain of the 14 STEC strains (Supplementary, Table S4). The fresh overnight culture of each STEC strain (0.1 mL) was mixed with 12 mL molten tryptic soy agar (TSA, Difco, Becton Dickinson, Sparks, MD, USA) and poured into a sterile Petri plate. After the strain-mixed agar solidified, 10 μL of the supernatant (obtained from the enrichment) were spotted on the TSA plate and incubated at 37 °C for 24 h. As a result, the supernatant was positive against the STEC O145 strain (RM10808) and further subjected to the phage purification process using a single-layer plaque assay as previously described, with minor modifications [26]. The single-layer plaque assay was conducted immediately after picking a plaque from the previous plaque assay plate for at least three runs, until the plaques were a similar size on the plate. After purification, the phage was propagated with the fresh overnight culture of the STEC O145 host (RM10808) in 40 mL TSB, supplemented with 10 mM of CaCl_2_ at 37 °C for 24 h. The propagated phage was centrifuged at 8000× *g* for 10 min and filtered through a 0.22-μm filter membrane to remove bacterial debris. The purified phages were subsequently concentrated using a 100 kDa cut-off Amicon Ultra-15 Centrifugal Filter Unit (Merck Millipore, Billerica, MA, USA) prior to downstream analyses such as transmission electron microscopy (TEM) and DNA extraction. 

### 4.3. Whole-Genome Sequencing and Genomic Analysis

Phage DNA was extracted using a phage DNA extraction kit from Norgen Biotek (Thorold, ON, Canada). The DNA library was prepared using a TruSeq Nano DNA Library Prep Kit (Illumina, San Deigo, CA, USA), and the final amplified libraries were quantified by a bioanalyzer (Agilent, Santa Clara, CA, USA) before sequencing. Approximately 6 million 2 × 250 bp pair-end sequence reads were generated using a MisSq Reagent Kit v3 (600-cycle) on the MiSeq platform (Illumina, San Deigo, CA, USA). The quality of raw sequence reads was first checked using FASTQC. The poor sequence reads (below Q30) were then trimmed using Trimmomatic (Galaxy Version 0.36.5, with the setting of average quality required for 30 (= Q30) to trim poor quality reads). A de novo assembly of the resulting quality reads was conducted using Unicycler Galaxy v0.4.6.0 (SPAdes) with default parameters. The final contig was annotated by Prokka (v.1.12.0) with default parameters, followed by manual characterization with PHASTER Webserver [41] and BLASTn against the viral nucleotide sequences obtained from National Center for Biotechnology Information (NCBI) with Geneious (v11.0.4). 

The annotated functions of the putative ORFs were confirmed using BLASTp. The prediction of tRNA in the phage genome was accomplished using tRNAscan-SE Search Server [42]. The phage termini and the possible packaging mechanism were predicted according to the in silico determination method proposed in PhageTerm [43]. The new phage sequence was subjected to a BLASTn search to obtain reference phage genomes with high nucleotide similarity from the NCBI database. These reference phage genomes were subjected to analysis with JSpeciesWS [44] to facilitate the taxonomic classification of the newly-isolated phage based on the degree of nucleotide sequence similarity [28]. The comparison of genome maps between phage Ro145clw and its reference genomes was visualized with the EasyFig visualization tool [45]. The presence of antibiotic-resistance genes in the phage genome was identified using the ResFinder (version 3.0) database [46]. Core gene analysis was conducted with CoreGenes3.5 Webserver, and genes with scores higher than 75 were considered core genes [47]. Comparative analysis of the core genes was conducted using the ClustalW algorithm for sequence alignment [48]. The phylogenetic tree was performed with MEGA 7 with the maximum composite likelihood method [49]. The reference phage genomes used in this study of *Escherichia* phage K1G (GenBank accession #GU196277), *Escherichia* phages ST2 (GenBank accession #MF153391), *Escherichia* phage G AB-2017 (GenBank accession #KY295895), and *Escherichia* phage P AB-2017 (GenBank accession #KY295898) were obtained from the NCBI database.

### 4.4. Biological Characteristics

#### 4.4.1. One-Step Growth Curve 

The experiment of a one-step growth curve was performed following the procedures described in Amarillas et al., with subtle modification [17]. The STEC O145 strain was inoculated in TSB and incubated at 37 °C overnight, then sub-cultured in 20 mL TSB at 37 °C until optical density at 600 nm (OD_600_) was 0.5. Subsequently, phage Ro145clw was added to a bacterial suspension at a MOI of 0.01, and the phage-bacteria mixture was kept at room temperature for 2 min for phage adsorption onto the bacterial cells. After the 2-min adsorption period, the mixture was centrifuged at 10,000× *g* for one minute at 4 °C. The supernatant was removed, and the phage titers were obtained to calculate the residual titers of the phages. The bacterial pellet containing infected strains was gently re-suspended in 20 mL TSB and incubated at 37 °C for 1 h, with sampling at 5-min intervals. At each sampling point, an aliquot of 1 mL of sample was obtained and centrifuged at 10,000× *g* for 30 s at 4 °C, followed by filtration through a 0.22-µm pore-size membrane filter, then a single-layer plaque assay, as described above in duplication. Simultaneously, an additional aliquot of 1 mL phage-infected culture was collected at each time point and treated with CHCl_3_. After homogenization for 2 min and centrifugation at 10,000× *g* for 2 min, the supernatant was obtained and subjected to serial dilutions prior to the single-layer plaque assay to determine the eclipse period. This entire experiment was conducted in three replications to estimate the burst size and latent period. The latent period was determined as the time that elapsed between the end of adsorption and the first release of phage progeny. The eclipse period was determined as the time period that elapsed between the end of adsorption and the appearance of phage particles within the bacterial cell. Burst size was calculated as the ratio of total number of phage particles produced to the initial number of infected bacterial cells during the latent period [50].

#### 4.4.2. Transmission Electronic Microscopy

The concentrated phage was used to examine the phage morphology with a transmission electron microscope (FEI Tecnai G_2_). An aliquot of 6 μL was placed on copper mesh PLECO grids (Ted Pella Inc., Redding, CA, USA) and left to set for 1 min at room temperature. Whatman filter paper was used to remove excessive phage lysate, followed by negative staining with an added 8 μL of 0.75% uranyl acetate (Sigma-Aldrich, Darmstadt, Germany) for 30 s staining at room temperature.

#### 4.4.3. Phage Stability

To examine pH susceptibility, 100 μL of the phage lysate was added to 900 μL of SM buffer with the final pH levels of 3.1, 5.1, 7.6, 9.2, and 10.5. Samples were incubated at 37 °C for 24 h. Viable phage particles were enumerated using the plaque assay. For the temperature test, phage lysate was added to SM buffer at a volume ratio of 1:9, and 1 mL of the phage solution was dispensed in sterile tubes prior to thermal treatments (65 and 73 °C). The phage titers were determined by the single-layer plaque assay every 10 min for 60 min. These temperatures were selected to evaluate the stability of Ro145clw in high-temperature processes such as pasteurization. The course of thermal treatment time (60 min) was selected to evaluate the short-term thermal stability of phage Ro145clw. For the stability of frozen storage, an aliquot of 500 μL phage lysate was mixed with glycerol at a final concentration of 25% and stored at −80 °C. 

### 4.5. Structural Protein Analysis

The purified and concentrated phage lysate was subjected to sodium dodecyl sulfate polyacrylamide gel electrophoresis (SDS-PAGE) using a 1D Biorad 12% TGX gel with Precision Plus MW standard marker (2 μL; Biorad, Hercules, CA, USA). Electrophoresis was performed at 100 V for 90 min. The gel was stained using an Imperial^TM^ Protein Stain (ThermoFisher, Waltham, MA, USA) containing a formulation of Coomassie brilliant blue R-250. In-gel digestions were conducted with Trypsin (Promega, Madison, WI, USA) using a Digest Pro digestion robot (Intavis, Köln, Germany). The robot was programmed to perform the in-gel digestion following methods that have been previously published [51,52]. Digested samples were subjected to nanoflow reversed-phase chromatography with an Eksigent NanoLC (Sciex, Framingham, MA, USA) using Picochip 105 mm columns packed with REPROSIL-Pur C18-AQ, 3 μM, 120A packing (New Objectives, Woburn, MA, USA). A 10 μL portion of each digested sample was injected with 2% acetonitrile in water with 0.1% formic acid, with a flow rate of 400 nL/min for 1 h. Elution solvents A and B were 2% acetonitrile in water and acetonitrile, respectively (each containing 0.1% formic acid). Sample elution began with 3% B, ramping up to 10% B at 10 min, then to 25% B at 40 min, then to 40% B at 58 min, returning to 3% B at 60 min. Mass spectral analyses were performed with an Orbitrap Elite (Thermo Fisher Scientific, Waltham, MA, USA), operated in positive ion mode using a top three data-dependent data acquisition method. Survey scans were collected at 60 K resolution in the Orbitrap detector. The top three most intense ions above the threshold of 30 K counts were subjected to collision-induced fragmentation (CID) with normalized collision energy set to 30. The resulting fragment ions were detected in the instrument’s linear trap. Dynamic exclusion of precursor ions was set to 6 s. Mascot software (Matrix Science, Boston, MA, USA) was used to match the tandem mass spectrometry (MS–MS) data to amino acid sequences derived from the nucleotide sequences that were obtained from the phage isolates.

### 4.6. Antimicrobial Activities

#### 4.6.1. Host Range and Efficiency of Plating 

After phage purification, the phage was subjected to the host range test against three non-pathogenic *E. coli*, 28 STEC and five *Salmonella* strains using the spot test assay as described above. For the spot test-positive strains, efficiency of plating (EOP) was used to determine productive infection by using phage particles produced against each susceptible strain in comparison to the phage particles produced against the primary host strain [53]. Fresh overnight cultures of the test strains and of the primary host strain were prepared in TSB at 37 °C for 18 h. After serial dilution, the phage lysates with four dilution factors (10^−3^ to 10^−7^) were subjected to the single-layer plaque assay separately against all test strains and the primary host strain. The plates were then incubated at 37 °C overnight. The experiment was conducted in three replications. EOP was calculated based on the average of plaque-forming units (PFU) against each test bacterium divided by the average of PFU against the primary bacterium used for isolation. Generally, if the EOP was 0.5 or more, it was classified as having a high phage-producing efficiency. An EOP above 0.1 but below 0.5 indicated a medium-producing efficiency; an EOP between 0.001 and 0.1 indicated a low-producing efficiency; any value under 0.001 represented inefficient phage production.

#### 4.6.2. Bacterial Challenge Assay

The bacterial challenge assay was conducted using a spectrophotometer to monitor bacterial growth treated with different concentrations of phage lysate as previously described, with minor modifications [54]. Prior to the experiment, a fresh overnight culture of *E. coli* O145:H28 was prepared in 5 mL of TSB and incubated at 37 °C for 18 h. Subsequently, the culture was pelleted down at 4000× *g* centrifugation and washed twice with the same volume of fresh TSB. After resuspension in TSB, the bacterial culture was diluted down to 1 × 10^5^ CFU/mL and further dispensed into a 96-well plate, with 200 μL per well; then, phage Ro145clw was added at MOIs of 1, 10, and 100. The plate was monitored in a plate reader with the temperature set at 37 °C. The OD_600_ reading was recorded every 30 min for 18 h. 

#### 4.6.3. Determination of Bacteriophage-Insensitive Mutant (BIM) Frequency

The emergence frequency of bacteriophage-insensitive mutant (BIM) was conducted by mixing appropriate volume of overnight cultures of STEC strains [*E. coli* O145:H28 (RM13514) and *E. coli* O145:NM (SJ23)] with phage Ro145clw at MOI of 100. The mixture was added with CaCl_2_ (10 mM) and MgSO_4_ (10mM) and then incubated at 37 °C for 10 min. After serial dilutions, the diluted bacterium-phage mixture was plated on MacConkey agar with a top thin layer of TSA (5 mL) and incubated at 37 °C overnight. BIM frequency was determined by dividing the number of surviving bacterial cells by the initial bacterial concentration. The experiments were conducted in 3 replications.

#### 4.6.4. Antimicrobial Activity Test in LB 

A fresh overnight culture of *E. coli* O145:H28 was prepared in 10 mL TSB at 37 °C for 18 h. An aliquot of 0.2 mL overnight culture was added to 18.8 mL LB (Invitrogen, Carlsbad, CA, USA) to obtain the final concentration at 1 × 10^7^ CFU/mL. One tube of the bacterial suspension was treated with phage lysates at 1 × 10^9^ PFU/mL (MOI 100). A control group was also prepared by adding the same volume of SM buffer to 20 mL of bacterial suspension. Both the control and treatment were incubated at 8 and 37 °C. At 0, 2, 4, 6, and 24 h of incubation, samples were serially diluted using sterile 0.1% peptone water, and an aliquot of 0.1 mL diluted sample was spread-plated on MacConkey agar plates (BD, Franklin Lakes, NJ, USA). The plates were incubated at 37 °C overnight, and colonies of bacteria were counted. The antimicrobial effects of the phage against *E. coli* O145:H28 (representing a low EOP) and *E*. *coli* O145: NM (high EOP) were compared using the same method as described at 25 °C. 

#### 4.6.5. Statistical Analysis

Experiments were performed with at least three individual repetitions. Bacterial colony counts and phage titers were calculated as CFU/mL or PFU/mL and logarithmically transformed for statistical analysis. Least squares mean (LSM) was performed to compare the means of phage titers using JMP^®^ (Version 12.0.1, SAS Institute Inc., Cary, NC, USA). One-way analysis of variance (ANOVA) with the statistical significance at 5% level was used to evaluate the effects of different pH on the recovery of phage titers. The Student’s *t*-test was used to evaluate the viable bacterial count between the control group and treatment group with the phage at each time point.

### 4.7. Nucleotide Sequence Accession Number

The genome sequence of *Escherichia* phage vB_EcoS-Ro145clw was deposited in GenBank under accession number MG852086. The raw sequence reads were submitted to the NCBI sequence read archive (SRA) with accession number PRJNA525899.

## 5. Conclusions

In this study, phage Ro145clw, with strong lytic infection against environmental STEC O145 strains, was shown to have a relatively short latent period (21 min) and a large burst size (192 phages per infected cell). The genomic data indicated the absence of unwanted genes including virulence genes, antibiotic-resistance genes, and lysogenic genes in the Ro145clw genome. Phage Ro145clw is resistant to adverse pH and temperature conditions; these features might be associated with the environment from which the phage was isolated. The antimicrobial effects of the phage against environmental STEC O145 strains were more prominent than against outbreak strains. These findings substantiate the potential biocontrol alternative of the phage Ro145clw to prevent the spread of STEC O145 in the pre-harvest environment. The genomic information for phage Ro145clw provides valuable insights into the diversity of the specific lytic phages against STEC strains. Future studies in using phage cocktails may be undertaken to improve the biocontrol effectiveness against outbreak STEC strains.

## Figures and Tables

**Table 1:** Host range and efficiency of plating (EOP) of phage Ro145clw against different serogroups of Shiga toxin-producing *Escherichia coli* (STEC) and *Salmonella* strains.

Strains	Strain Ref. No.	EOP ^α^
STEC O26	*E. coli* O26:H18 (RM17857), *E. coli* O26:H- (RM18118) *E. coli* O26:H- (RM18132), *E. coli* O26:H- (RM17133)	R *
STEC O103	*E. coli* O103:H2 (RM12551), *E. coli* O103:H2 (RM13322) *E. coli* O103:H- (RM8356), *E. coli* O103:H- (RM10744)	R
STEC O121	*E. coli* O121:H19 (RM10046), *E. coli* O121:H19 (RM10068) *E. coli* O121:H- (RM8082), *E. coli* O121:H- (RM12997)	R
STEC O111	*E. coli* O111:H2 (RM13483), *E. coli* O111:H- (RM13789) *E. coli* O111:H- (RM11765), *E. coli* O111:H8 (RM14488)	R
STEC O145	*E. coli* O145:H+ (RM8732)	0.73
	*E. coli* O145:H+ (RM11691)	0.64
	*E. coli* O145:H+ (RM12367)	0.67
	*E. coli* O145:H- (RM10808)	H ^
	*E. coli* O145:H28 (RM9872)	0.59
	*E. coli* O145:H28 (RM13514)	<0.001
	*E. coli* O145:H28 (RM13516)	<0.001
	*E. coli* O145:H28 (RM12761)	<0.001
	*E. coli* O145:H28 (RM12581)	<0.001
	*E. coli* O145:NM (SJ23)	1.05
	*E. coli* O145:H- (94-0491)	0.29
STEC O45	*E. coli* O45:H- (RM10729), *E. coli* O45:H- (RM13726) *E. coli* O45:H- (RM13745), *E. coli* O45:H- (RM13752)	R
STEC O157	*E. coli* O157:H7 (RM18959), *E. coli* O157:H7 (RM18961) *E. coli* O157:H7 (RM18972), *E. coli* O157:H7 (RM18974) *E. coli* O157:H7 (ATCC 43888)	R
*Salmonella*	*Salmonella* Montevideo 51, *Salmonella* Newport H1073 *Salmonella* Heidelberg 45955, *Salmonella* Enteritidis PT30 *Salmonella* Typhimurium 14028	R

^α^ EOP was conducted on spot test-positive strains and is presented with a value that was calculated by the ratio of phage titer on test bacterium relative to the phage titer on the primary bacterium used for isolation. High production efficiency is EOP ≥ 0.5, medium production efficiency is 0.5 > EOP ≥ 0.1, low production efficiency is 0.1 > EOP > 0.001, and inefficiency of phage production is EOP ≤ 0.001. * R denotes no lysis in the spot test assay. ^ H was the primary bacterial strain used for isolation.

**Table 2:** Structural proteins of phage Ro145clw, identified by matrix assisted laser desorption ionization-time of flight (MALDI-TOF) mass spectrometry.

Gel Band	ORF	Putative Function	Sequence Coverage (%)	No. of Peptides	Predicted Mass (kDa)
A	20	Putative tail assembly chaperone	11	11	95.5
B	17	Tape measure protein	29	16	80.8
C	62	Putative structural protein	22	8	54.7
D	9	Tail protein	25	5	40.9
E	1	Major capsid protein	62	12	37.8

## Supplementary Materials

The following are available online at https://www.mdpi.com/2079-6382/8/2/74/s1: Figure S1: Bacterial challenge assay of the phage Ro145clw against outbreak strain *E. coli* O145:H28; Table S1: List of annotated ORFs with the size, location, and predicted functions in the genome of Ro145clw; Table S2: Storage evaluation of phage Ro145clw in 25% glycerol at –80 °C for five months; Table S3: Comparison of the antimicrobial activities of phage Ro145clw (MOI of 100) against different EOP bacterial strains (initial concentration = 7 log CFU/mL) in LB at 25 °C; Table S4: Bacterial strains including *E. coli* and *Salmonella* were used in the present study for either phage isolation or the host range test of the isolated phage.

## References

[1] Tarr P.I., Gordon C.A., Chandler W.L. (2005). Shiga-toxin-producing *Escherichia coli* and haemolytic uraemic syndrome. Lancet.

[2] Riley L.W., Remis R.S., Helgerson S.D., McGee H.B., Wells J.G., Davis B.R., Hebert R.J., Olcott E.S., Johnson L.M., Hargrett N.T. (1983). Hemorrhagic colitis associated with a rare *Escherichia coli* serotype. N. Engl. J. Med..

[3] Scallan E., Hoekstra R.M., Angulo F.J., Tauxe R.V., Widdowson M.-A., Roy S.L., Jones J.L., Griffin P.M. (2011). Foodborne illness acquired in the United States–Major pathogens. Emerg. Infect. Dis..

[4] Karmali M.A. (2017). Emerging Public Health Challenges of Shiga Toxin-Producing *Escherichia coli* Related to Changes in the Pathogen, the Population, and the Environment. Clin. Infect. Dis..

[5] Herman K.M., Hall A.J., Gould L.H. (2015). Outbreaks attributed to fresh leafy vegetables, United States, 1973–2012. Epidemiol. Infect..

[6] Brooks J.T., Sowers E.G., Wells J.G., Greene K.D., Griffin P.M., Hoekstra R.M., Strockbine N.A. (2005). Non-O157 Shiga toxin-producing *Escherichia coli* infections in the United States, 1983–2002. J. Infect. Dis..

[7] Luna-Gierke R.E., Griffin P.M., Gould L.H., Herman K., Bopp C.A., Strockbine N., Mody R.K. (2014). Outbreaks of non-O157 Shiga toxin-producing *Escherichia coli* infection: USA. Epidemiol. Infect..

[8] Taylor E.V., Nguyen T.A., Machesky K.D., Koch E., Sotir M.J., Bohm S.R., Folster J.P., Bokanyi R., Kupper A., Bidol S.A. (2013). Multistate outbreak of *Escherichia coli* O145 infections associated with romaine lettuce consumption, 2010. J. Food Prot..

[9] Rivero M.A., Passucci J.A., Rodriguez E.M., Parma A.E. (2010). Role and clinical course of verotoxigenic *Escherichia coli* infections in childhood acute diarrhoea in Argentina. J. Med. Microbiol..

[10] De Schrijver K., Buvens G., Posse B., Van den Branden D., Oosterlynck O., De Zutter L., Eilers K., Pierard D., Dierick K., Van Damme-Lombaerts R. (2008). Outbreak of verocytotoxin-producing *E. coli* O145 and O26 infections associated with the consumption of ice cream produced at a farm, Belgium, 2007. Euro Surveill..

[11] Carter M.Q., Quinones B., He X., Zhong W., Louie J.W., Lee B.G., Yambao J.C., Mandrell R.E., Cooley M.B. (2015). An Environmental Shiga Toxin-Producing *Escherichia coli* O145 Clonal Population Exhibits High-Level Phenotypic Variation That Includes Virulence Traits. Appl. Environ. Microbiol..

[12] Liao Y.T., Brooks J.C., Martin J.N., Echeverry A., Loneragan G.H., Brashears M.M. (2015). Antimicrobial interventions for O157:H7 and non-O157 Shiga toxin-producing *Escherichia coli* on beef subprimal and mechanically tenderized steaks. J. Food Prot..

[13] Hatfull G.F. (2008). Bacteriophage genomics. Curr. Opin. Microbiol..

[14] Clokie M.R.J., Millard A.D., Letarov A.V., Heaphy S. (2011). Phages in nature. Bacteriophage.

[15] Hagens S., Loessner M.J. (2010). Bacteriophage for biocontrol of foodborne pathogens: Calculations and considerations. Curr. Pharm. Biotechnol..

[16] Snyder A.B., Perry J.J., Yousef A.E. (2016). Developing and optimizing bacteriophage treatment to control enterohemorrhagic *Escherichia coli* on fresh produce. Int. J. Food Microbiol..

[17] Amarillas L., Chaidez C., Gonzalez-Robles A., Lugo-Melchor Y., Leon-Felix J. (2016). Characterization of novel bacteriophage phiC119 capable of lysing multidrug-resistant Shiga toxin-producing *Escherichia coli* O157:H7. PeerJ.

[18] Svab D., Falgenhauer L., Rohde M., Szabo J., Chakraborty T., Toth I. (2018). Identification and Characterization of T5-Like Bacteriophages Representing Two Novel Subgroups from Food Products. Front. Microbiol..

[19] Tolen T.N., Xie Y., Hairgrove T.B., Gill J.J., Taylor T.M. (2018). Evaluation of Commercial Prototype Bacteriophage Intervention Designed for Reducing O157 and Non-O157 Shiga-Toxigenic *Escherichia coli* (STEC) on Beef Cattle Hide. Foods.

[20] Catalao M.J., Gil F., Moniz-Pereira J., Sao-Jose C., Pimentel M. (2013). Diversity in bacterial lysis systems: Bacteriophages show the way. FEMS Microbiol. Rev..

[21] Baig A., Colom J., Barrow P., Schouler C., Moodley A., Lavigne R., Atterbury R. (2017). Biology and Genomics of an Historic Therapeutic *Escherichia coli* Bacteriophage Collection. Front. Microbiol..

[22] Summer E.J., Berry J., Tran T.A.T., Niu L., Struck D.K., Young R. (2007). Rz/Rz1 lysis gene equivalents in phages of Gram-negative hosts. J. Mol. Biol..

[23] Oliveira L., Tavares P., Alonso J.C. (2013). Headful DNA packaging: Bacteriophage SPP1 as a model system. Virus Res..

[24] Wang J., Niu Y.D., Chen J., Anany H., Ackermann H.W., Johnson R.P., Ateba C.N., Stanford K., McAllister T.A. (2015). Feces of feedlot cattle contain a diversity of bacteriophages that lyse non-O157 Shiga toxin-producing *Escherichia coli*. Can. J. Microbiol..

[25] Litt P.K., Saha J., Jaroni D. (2018). Characterization of Bacteriophages Targeting Non-O157 Shiga Toxigenic *Escherichia coli*. J. Food Prot..

[26] Liao Y.-T., Quintela I.A., Nguyen K., Salvador A., Cooley M.B., Wu V.C.H. (2018). Investigation of prevalence of free Shiga toxin-producing *Escherichia coli* (STEC)-specific bacteriophages and its correlation with STEC bacterial hosts in a produce-growing area in Salinas, California. PLoS ONE.

[27] Endersen L., Guinane C.M., Johnston C., Neve H., Coffey A., Ross R.P., McAuliffe O., O’Mahony J. (2015). Genome analysis of *Cronobacter* phage vB_CsaP_Ss1 reveals an endolysin with potential for biocontrol of Gram-negative bacterial pathogens. J. Gen. Virol..

[28] Adriaenssens E.M., Brister J.R. (2017). How to Name and Classify Your Phage: An Informal Guide. Viruses.

[29] Hatfull G.F., Hendrix R.W. (2011). Bacteriophages and their genomes. Curr. Opin. Virol..

[30] Peng Q., Yuan Y. (2018). Characterization of a newly isolated phage infecting pathogenic *Escherichia coli* and analysis of its mosaic structural genes. Sci. Rep..

[31] Wietzorrek A., Schwarz H., Herrmann C., Braun V. (2006). The genome of the novel phage Rtp, with a rosette-like tail tip, is homologous to the genome of phage T1. J. Bacteriol..

[32] Hoyles L., Murphy J., Neve H., Heller K.J., Turton J.F., Mahony J., Sanderson J.D., Hudspith B., Gibson G.R., McCartney A.L. (2015). *Klebsiella pneumoniae* subsp. *pneumoniae*-bacteriophage combination from the caecal effluent of a healthy woman. PeerJ.

[33] Xu Y., Yu X., Gu Y., Huang X., Liu G., Liu X. (2018). Characterization and Genomic Study of Phage vB_EcoS-B2 Infecting Multidrug-Resistant *Escherichia coli*. Front. Microbiol..

[34] Nilsson A.S. (2014). Phage therapy–Constraints and possibilities. Upsala J. Med. Sci..

[35] Son H.M., Duc H.M., Masuda Y., Honjoh K.I., Miyamoto T. (2018). Application of bacteriophages in simultaneously controlling *Escherichia coli* O157:H7 and extended-spectrum beta-lactamase producing *Escherichia coli*. Appl. Microbiol. Biotechnol..

[36] Wang J., Niu Y.D., Chen J., McAllister T.A., Stanford K. (2015). Complete Genome Sequence of *Escherichia coli* O145:NM Bacteriophage vB_EcoM_AYO145A, a New Member of O1-Like Phages. Genome Announc..

[37] Merabishvili M., Vervaet C., Pirnay J.-P., De Vos D., Verbeken G., Mast J., Chanishvili N., Vaneechoutte M. (2013). Stability of *Staphylococcus aureus* phage ISP after freeze-drying (lyophilization). PLoS ONE.

[38] Geagea H., Labrie S.J., Subirade M., Moineau S. (2018). The Tape Measure Protein Is Involved in the Heat Stability of *Lactococcus lactis* Phages. Appl. Environ. Microbiol..

[39] Hudson J.A., Billington C., Cornelius A.J., Wilson T., On S.L.W., Premaratne A., King N.J. (2013). Use of a bacteriophage to inactivate *Escherichia coli* O157:H7 on beef. Food Microbiol..

[40] Tomat D., Migliore L., Aquili V., Quiberoni A., Balagué C. (2013). Phage biocontrol of enteropathogenic and shiga toxin-producing *Escherichia coli* in meat products. Front. Cell. Infect. Microbiol..

[41] Arndt D., Grant J.R., Marcu A., Sajed T., Pon A., Liang Y., Wishart D.S. (2016). PHASTER: A better, faster version of the PHAST phage search tool. Nucleic Acids Res..

[42] Lowe T.M., Chan P.P. (2016). tRNAscan-SE On-line: Integrating search and context for analysis of transfer RNA genes. Nucleic Acids Res..

[43] Garneau J.R., Depardieu F., Fortier L.C., Bikard D., Monot M. (2017). PhageTerm: A tool for fast and accurate determination of phage termini and packaging mechanism using next-generation sequencing data. Sci. Rep..

[44] Richter M., Rossello-Mora R., Oliver Glockner F., Peplies J. (2016). JSpeciesWS: A web server for prokaryotic species circumscription based on pairwise genome comparison. Bioinformatics.

[45] Sullivan M.J., Petty N.K., Beatson S.A. (2011). Easyfig: A genome comparison visualizer. Bioinformatics.

[46] Zankari E., Cosentino S., Vestergaard M., Rasmussen S., Lund O., Aarestrup F.M., Larsen M.V. (2012). Identification of acquired antimicrobial resistance genes. J. Antimicrob. Chemother..

[47] Mahadevan P., King J.F., Seto D. (2009). CGUG: In silico proteome and genome parsing tool for the determination of “core” and unique genes in the analysis of genomes up to ca. 1.9 Mb. BMC Res. Methods.

[48] McWilliam H., Li W., Uludag M., Squizzato S., Park Y.M., Buso N., Cowley A.P., Lopez R. (2013). Analysis Tool Web Services from the EMBL-EBI. Nucleic Acids Res..

[49] Tamura K., Stecher G., Peterson D., Filipski A., Kumar S. (2013). MEGA6: Molecular Evolutionary Genetics Analysis version 6.0. Mol. Biol. Evol..

[50] Adams M.H. (1959). Bacteriophage.

[51] Shevchenko A., Tomas H., Havlis J., Olsen J.V., Mann M. (2006). In-gel digestion for mass spectrometric characterization of proteins and proteomes. Nat. Protoc..

[52] Shevchenko A., Wilm M., Vorm O., Mann M. (1996). Mass spectrometric sequencing of proteins silver-stained polyacrylamide gels. Anal. Chem..

[53] Mirzaei M.K., Nilsson A.S. (2015). Correction: Isolation of phages for phage therapy: A comparison of spot tests and efficiency of plating analyses for determination of host range and efficacy. PLoS ONE.

[54] Fong K., LaBossiere B., Switt A.I.M., Delaquis P., Goodridge L., Levesque R.C., Danyluk M.D., Wang S. (2017). Characterization of Four Novel Bacteriophages Isolated from British Columbia for Control of Non-typhoidal *Salmonella in Vitro* and on Sprouting Alfalfa Seeds. Front. Microbiol..

