# Supplementary material for: Characterization of a Lytic Bacteriophage as an Antimicrobial Agent for Biocontrol of Shiga Toxin-Producing Escherichia coli O145 Strains
Source: Antibiotics (Basel). 2019 Jun 5;8(2):74. doi: 10.3390/antibiotics8020074 (PMC6627115; doi:10.3390/antibiotics8020074)
Supplement: Supplementary file 1 [file antibiotics-08-00074-s001.pdf]

Supplementary Materials

Table S1. List of annotated ORFs with the size, location and predicted functions in the genome of Ro145clw.

ORFs	Predicted Function[a]	Functional Category[b]	Starting bp	Ending bp	Length of ORF (bp)	Direction
1	Major capsid protein CDS	Structural protein	299	1348	1050	forward
2	hypothetical protein CDS	n/a	1408	1707	300	forward
3	Decoration protein CDS	Structural protein	1720	2070	351	forward
4	hypothetical protein CDS	n/a	2107	2286	180	forward
5	hypothetical protein CDS	n/a	2290	2802	513	forward
6	hypothetical protein CDS	n/a	2805	3419	615	forward
7	hypothetical protein CDS	n/a	3419	3778	360	forward
8	hypothetical protein CDS	n/a	4173	4586	414	forward
9	Tail protein CDS	Structural protein	4589	5755	1167	forward
10	hypothetical protein CDS	n/a	5781	6269	489	reverse
11	hypothetical protein CDS	n/a	6313	6543	231	reverse
12	hypothetical protein CDS	n/a	6557	7027	471	reverse
13	Putative calcineurin-like phosphoesterase superfamily domain protein CDS	DNA replication	7024	8154	1131	reverse
14	hypothetical protein CDS	n/a	8193	8384	192	reverse
15	hypothetical protein CDS	n/a	8552	8971	420	forward
16	hypothetical protein CDS	n/a	9070	9336	267	forward
17	Tape measure protein CDS	Structural protein	9329	11599	2271	forward
18	hypothetical protein CDS	n/a	12094	12609	516	forward
19	hypothetical protein CDS	n/a	12606	12971	366	forward

20	Putative tail assembly chaperone CDS	Structural protein	12962	15517	2556	forward
21	Putative tail fiber protein CDS	Structural protein	15530	17620	2091	forward
22	hypothetical protein CDS	n/a	17649	17789	141	reverse
23	hypothetical protein CDS	n/a	17786	17974	189	reverse
24	putative helicase CDS	DNA replication	17971	19395	1425	reverse
25	hypothetical protein CDS	n/a	19648	19842	195	reverse
26	hypothetical protein CDS	n/a	19842	20045	204	reverse
27	hypothetical protein CDS	n/a	20078	20362	285	reverse
28	hypothetical protein CDS	n/a	20362	20511	150	reverse
29	hypothetical protein CDS	n/a	20594	20725	132	reverse
30	Putative thermostable DNA polymerase I CDS	DNA replication	20738	22915	2178	reverse
31	hypothetical protein CDS	n/a	22974	23603	630	reverse
32	hypothetical protein CDS	n/a	23690	23845	156	reverse
33	Putative PD-(D/E)XK nuclease superfamily protein CDS	DNA replication	23842	25083	1242	reverse
34	hypothetical protein CDS	n/a	25055	25351	297	reverse
35	hypothetical protein CDS	n/a	25395	25895	501	reverse
36	Transcriptional repressor DicA CDS	DNA replication	26021	26236	216	forward
37	Putative helicase-primase CDS	DNA replication	26254	28494	2241	reverse
38	hypothetical protein CDS	n/a	28553	28786	234	reverse
39	hypothetical protein CDS	n/a	28783	28953	171	reverse
40	hypothetical protein CDS	n/a	29448	29633	186	forward
41	hypothetical protein CDS	n/a	29633	29887	255	forward

42	hypothetical protein CDS	n/a	30096	30305	210	forward
43	hypothetical protein CDS	n/a	30430	30606	177	forward
44	hypothetical protein CDS	n/a	30673	30822	150	forward
45	hypothetical protein CDS	n/a	30812	31081	270	forward
46	hypothetical protein CDS	n/a	31101	31211	111	forward
47	hypothetical protein CDS	n/a	31217	31579	363	forward
48	hypothetical protein CDS	n/a	31576	31779	204	forward
49	hypothetical protein CDS	n/a	31782	31964	183	forward
50	hypothetical protein CDS	n/a	31964	32257	294	forward
51	Putative holin-like class II CDS	Cell lysis	32333	32641	309	forward
52	Putative holin-like class I CDS	Cell lysis	32634	32906	273	forward
53	Endolysin CDS	Cell lysis	32884	33372	489	forward
54	hypothetical protein CDS	n/a	33628	33777	150	forward
55	hypothetical protein CDS	n/a	33774	33944	171	forward
56	hypothetical protein CDS	n/a	33941	34186	246	forward
57	hypothetical protein CDS	n/a	34218	34457	240	forward
58	hypothetical protein CDS	n/a	34992	35228	237	forward
59	hypothetical protein CDS	n/a	35225	35407	183	forward
60	hypothetical protein CDS	n/a	35627	36172	546	forward
61	putative terminase CDS	Packaging	36169	37419	1251	forward
62	Putative structural protein CDS	Structural protein	37432	38907	1476	forward
63	Putative head protein CDS	Structural protein	38904	39959	1056	forward
64	Putative tail protein CDS	Structural protein	39959	40420	462	forward
65	hypothetical protein CDS	n/a	40449	40673	225	reverse
66	putative spanin protein CDS	Cell lysis	41048	41434	387	forward
67	putative spanin protein CDS	Cell lysis	41397	41543	147	forward

[a] CDS means coding sequence.

[b] n/a means the ORFs do not have any functional category.

Table S2. Storage evaluation of phage Ro145clw in 25% glycerol at -80°C for 5 months.

Temperature	Before storage (Log PFU/ml)*	After storage (Log PFU/ml)$^{\alpha}$
-80°C	10.43	10.22

* Phage titers were obtained prior to addition of glycerol for frozen storage.

$^{\alpha}$ Phage titers were obtained right after thawing the phage solution.

Table S3. Comparison of the antimicrobial activities of phage Ro145clw (MOI of 100) against different EOP bacterial strains* (initial concentration= 7 log CFU/ml) in LB broth at 25°C.

**Incubation time (h)**	**Bacterial reduction (Log CFU/ml)$^{\alpha}$**	
	E. coli O145:NM	*E. coli* O145:H28
0 h	0.40 ± 0.13	0.27 ± 0.05
6 h	6.20 ± 0.26	3.51 ± 0.30
24 h	1.65 ± 0.07	1.05 ± 0.16

* Two strains, including *E. coli* O145:NM and *E. coli* O145:H28 (RM13514), were used; The *E. coli* O145:NM strain has EOP=1.05 and *E. coli* O145:H28 strain has EOP < 0.001.

$^{\alpha}$ Reduction data are represented as means ± standard deviation and consist of three replicates compared to the bacterial population (log CFU/ml) recovered from untreated controls (no phages) at each time point.

Table S4. Bacterial strains, including *E. coli* and *Salmonella*, used in current study for either phage isolation or host range test of the isolated phage.

Internal Ref. No.	Strain	Source[a]	Serogroup	*eae*	*Stx1*	*Stx2*	Note[b]
RM17857	*E. coli* O26:H18	water	26	-	+	-	
RM18118	*E. coli* O26	water	26	+	+	-	
RM18132	*E. coli* O26	water	26	+	+	-	Host panel
RM17133	*E. coli* O26	water	26	-	+	-	Host panel
RM12551	*E. coli* O103:H2	water	103	+	+	-	
RM13322	*E. coli* O103:H2	cattle feces	103	+	+	-	Host panel
RM8356	*E. coli* O103	water	103	-	-	+	
RM10744	*E. coli* O103	cattle feces	103	+	-	+	Host panel
RM10046	*E. coli* O121:H19	cattle feces	121	+	-	+	
RM10068	*E. coli* O121:H19	trough water	121	+	-	+	
RM8082	*E. coli* O121	cattle feces	121	-	+	+	Host panel
RM12997	*E. coli* O121	pig feces	121	-	+	-	Host panel
RM13483	*E. coli* O111:H2	cattle feces	111	+	+	-	
RM13789	*E. coli* O111	water	111	+	+	-	
RM11765	*E. coli* O111	water	111	+	+	-	Host panel
RM14488	*E. coli* O111	water	111	+	-	+	Host panel
RM8732	*E. coli* O145	water	145	+	+	-	
RM11691	*E. coli* O145	water	145	+	+	-	
RM12367	*E. coli* O145	water	145	+	-	+	Host panel
RM10808	*E. coli* O145	cattle feces	145	+	+	-	Host panel
RM9872	*E. coli* O145:H28	Cattle feces	145	-	-	+	
RM13514	*E. coli* O145:H28	Outbreak strain	145	+	-	+	
RM13516	*E. coli* O145:H28	Outbreak strain	145	+	-	+	
RM12761	*E. coli* O145:H28	Outbreak strain	145	+	-	+	

RM12581	*E. coli* O145:H28	Outbreak strain	145	+	-	+	
SJ23	*E. coli* O145:NM	environment	145	-	+	+	
94-0491	*E. coli* O145:H-	environment	145	-	+	+	
RM10729	*E. coli* O45	cattle	45	-	+	-	Host panel
RM13726	*E. coli* O45	cattle	45	-	+	-	
RM13745	*E. coli* O45	cattle	45	-	+	-	
RM13752	*E. coli* O45	cattle	45	-	+	-	Host panel
RM18959	*E. coli* O157:H7	water	O157	+	-	+	Host panel
RM18961	*E. coli* O157:H7	water	O157	+	-	+	
RM18972	*E. coli* O157:H7	water	O157	+	+	+	
RM18974	*E. coli* O157:H7	water	O157	+	-	+	Host panel
DH5α	Generic *E. coli*						Host panel
ATCC 13706	Generic *E. coli*						Host panel
ATCC 43888	*E. coli* O157:H7 (without *stx* gene)		O157				Host panel
n/a	*Salmonella* Montevideo 51						
n/a	*Salmonella* Newport H1073						
n/a	*Salmonella* Heidelberg 45955						
n/a	*Salmonella* Enteritidis PT30						
n/a	*Salmonella* Typhimurium 14028						

[a] The source is the type of environmental samples where the strain was originally isolated from.

[b] Host panel means the strains were used for the enrichment of the environmental samples for phage isolation.

"-" is the negative and "+" is positive of the PCR results.

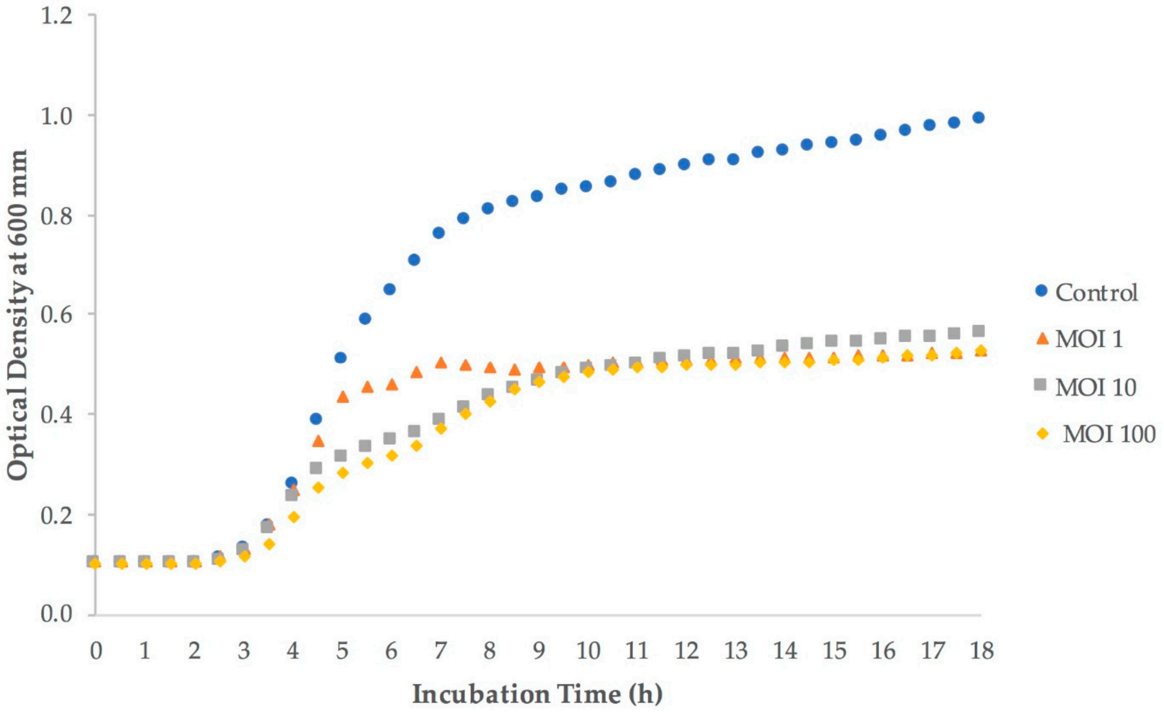

Figure S1. Bacterial challenge assay of the *E. coli* O145:H28 strain (RM13514) treated with phage Ro145clw in 96-well plate. The OD_{600} was measured by spectrophotometer every 0.5 h for the period of 18-h incubation at 37°C. The control (circles) contains only bacterial culture. MOI 1 (triangles) contains bacterial culture treated with the equal concentration of phage Ro145clw; MOI 10 (squares) contains bacterial culture treated with 10-fold more concentration of the phage; MOI 100 (diamonds) contains bacterial culture treated with 100-fold more concentration of the phage.
